# Impact of Adjuvant Chemotherapy in Patients With Curatively Resected Stage IV Colorectal Cancer

**DOI:** 10.1097/MD.0000000000000696

**Published:** 2015-05-01

**Authors:** Hirotoshi Kobayashi, Kenjiro Kotake, Kenichi Sugihara

**Affiliations:** From the Center for Minimally Invasive Surgery (HK); Department of Surgical Oncology, Tokyo Medical and Dental University, Bunkyo-ku, Tokyo (HK, KS); and Department of Surgery, Tochigi Cancer Center, Utsunomiya, Tochigi, Japan (KK).

## Abstract

The aim of this study was to investigate the impact of adjuvant chemotherapy on survival of patients who had curative resection for stage IV colorectal cancer.

The efficacy of adjuvant chemotherapy after curative resection for stage IV colorectal cancer remains unclear.

The database of 3695 patients with stage IV colorectal cancer between 1991 and 2007 collected from 16 member hospitals of the Japanese Society for Cancer of the Colon and Rectum was used for this investigation. The survivals of patients with and without adjuvant chemotherapy after curative resection for stage IV colorectal cancer were evaluated using a propensity score matching method.

The data of 689 patients who underwent curative resection for both primary and synchronous metastatic tumors were extracted from the database and used for analysis in this study. The 5-year overall survival rates of the patients with and without adjuvant chemotherapy were 41.8% and 33.9%, respectively. A Cox proportional hazards model showed that adjuvant chemotherapy (*P* = 0.0042), regional lymph node metastasis (*P* < 0.0001), and peritoneal metastasis (P = 0.0006) were independent factors for overall survival. In the propensity score-matched cohort, patients with adjuvant chemotherapy had better overall survival than those without (*P* = 0.026).

The present study demonstrated that adjuvant chemotherapy improved overall survival after curative resection for stage IV colorectal cancer. The efficacy of each chemotherapeutic regimen in the adjuvant setting for stage IV colorectal cancer should be clarified in the future.

## INTRDUCTION

Colorectal cancer is the third leading cause of cancer deaths in Japan, and the number of persons affected has been increasing rapidly.^1,2^ Both synchronous hematogenous and peritoneal metastases are poor prognostic factors in patients with colorectal cancer and are classified into stage IV in the TNM staging system.^3^ A number of studies have established the impact of adjuvant chemotherapy on patients with curatively resected stage III colorectal cancer.^4–7^ However, few studies have shown a benefit of adjuvant chemotherapy after curative resection for stage IV colorectal cancer.^8,9^

The aim of this study was to investigate the impact of adjuvant chemotherapy on patients with curatively resected stage IV colorectal cancer.

## METHODS

### Patients

The data of the 3965 patients with stage IV colorectal cancer who underwent surgery between 1991 and 2007 were collected from 16 member institutions of the Japanese Society for Cancer of the Colon and Rectum (JSCCR) in Japan. This study was approved by the ethics committee of the JSCCR and each institutional review board. Of these patients, 2954 had operative information regarding curability. Among these, 840 patients underwent curative resection for both primary and metastatic tumors; 689 had detailed information, and their data were further analyzed.

### Parameters

The parameters used in this study were: age, sex, location of primary tumor, histologic type, depth of tumor invasion (T-category), lymph node metastasis (N-category), liver metastasis, hematogenous metastasis other than liver metastasis, peritoneal metastasis, and operation periods. Tumor location was classified into either right colon or left colon and rectum. Right colon included cecum, ascending colon, and transverse colon.

### Statistical Analysis

Associations between adjuvant chemotherapy for stage IV colorectal cancer and categorical parameters were analyzed using the *χ*^2^ test. The actuarial survival after curative surgery was determined from Kaplan–Meier curves. The overall survival in each group was compared using the Wilcoxon test. The independent prognostic factors in patients with curative resection for stage IV colorectal cancer were determined using the Cox proportional hazards model.

Thereafter, pairwise 1:1 propensity score matching, including logistic regression, was used to reduce the effects of non-random assignment of patients to adjuvant chemotherapy. The propensity score matching method has been used to reduce potential confounding caused by unbalanced covariates.^10^ In short, by multivariate logistic regression analysis, the propensity score for adjuvant chemotherapy was determined. Patients with and without adjuvant chemotherapy were matched by greedy matching without replacement.

Data were analyzed statistically using SPSS 22 software (IBM, Armonk, NY). The data are expressed as numbers of patients and ratios (%) or means ± standard deviation. Statistical significance was established at *P* < 0.05 for all results.

## RESULTS

### Patients’ Characteristics

The patients’ characteristics for the entire cohort are shown in Table 1. The median age of the patients with and without adjuvant chemotherapy was 62 and 66 years, respectively. Among the 10 parameters, there were significant differences in age (*P* < 0.0001), histologic type (*P* = 0.015), depth of tumor invasion (*P* = 0.033), and distant metastasis other than liver (*P* = 0.0065) between patients with and without adjuvant chemotherapy after curative resection for stage IV colorectal cancer.

**TABLE 1:**
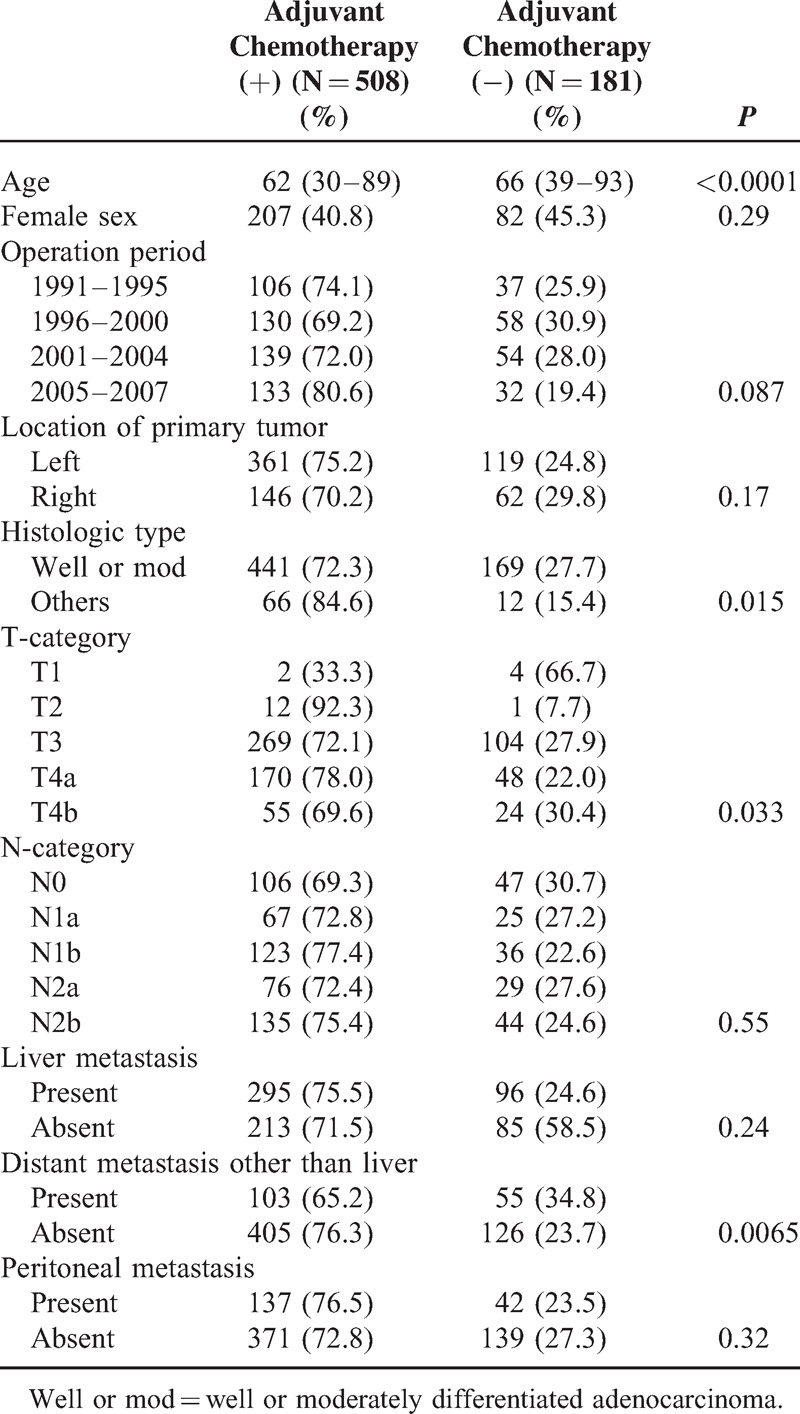
Characteristics of the Entire Cohort (689 Patients)

### Adjuvant Chemotherapy

Patients who underwent surgery before 2005 received fluorouracil-based adjuvant chemotherapy, while only those who underwent surgery in 2005 or later received cytotoxic adjuvant chemotherapy including FOLFOX, because oxaliplatin was approved in 2005 in Japan.

### Survival

**FIGURE 1:**
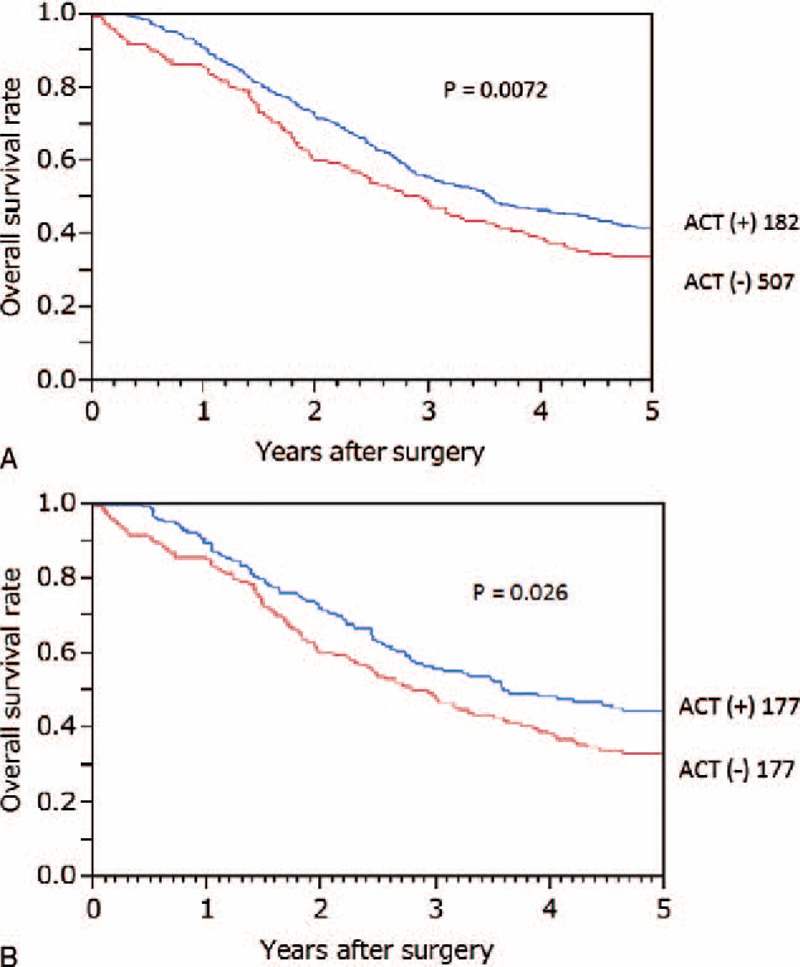
Overall survival curves of patients with and without adjuvant chemotherapy after curative resection for stage IV colorectal cancer in the entire cohort (A, *P* = 0.0072) and in the propensity score-matched cohort (B, *P* = 0.026). ACT = adjuvant chemotherapy.

The median follow-up period of the entire cohort was 2.8 (0–19.5) years. The median survival time of patients with and without adjuvant chemotherapy was 2.8 and 2.4 years, respectively (*P* = 0.039). Overall survival differed significantly between patients with and without adjuvant chemotherapy (*P* = 0.0072). The 5-year overall survival rates of the entire cohort with and without adjuvant chemotherapy were 44.4% and 32.4%, respectively (Figure 1A). The 5-year overall survival rates in each operation period (1991–1995, 1996–2000, 2001–2004, and 2005–2007) were 34.2%, 37.6%, 34.4%, and 47.3%, respectively (*P* = 0.023). The 5-year overall survival rates of patients with and without adjuvant chemotherapy in each period were 36.3%, 31.3% (*P* = 0.88); 42.9%, 33.7% (*P* = 0.41); 36.3%, 32.7% (*P* = 0.15); and 72.7%, 12.4% (*P* = 0.029).

### Prognostic Factors

Histologic type (*P* = 0.014), adjuvant chemotherapy (*P* = 0.031), depth of tumor invasion (*P* < 0.0001), regional lymph node metastasis (*P* < 0.0001), liver metastasis (*P* = 0.0055), and peritoneal metastasis (*P* < 0.0001) were associated with prognosis (Table 2). Among these factors, adjuvant chemotherapy (*P* = 0.0042), regional lymph node metastasis (*P* < 0.0001), and peritoneal metastasis (*P* = 0.0006) were independent factors for overall survival using Cox proportional hazards models (Table 2).

**TABLE 2:**
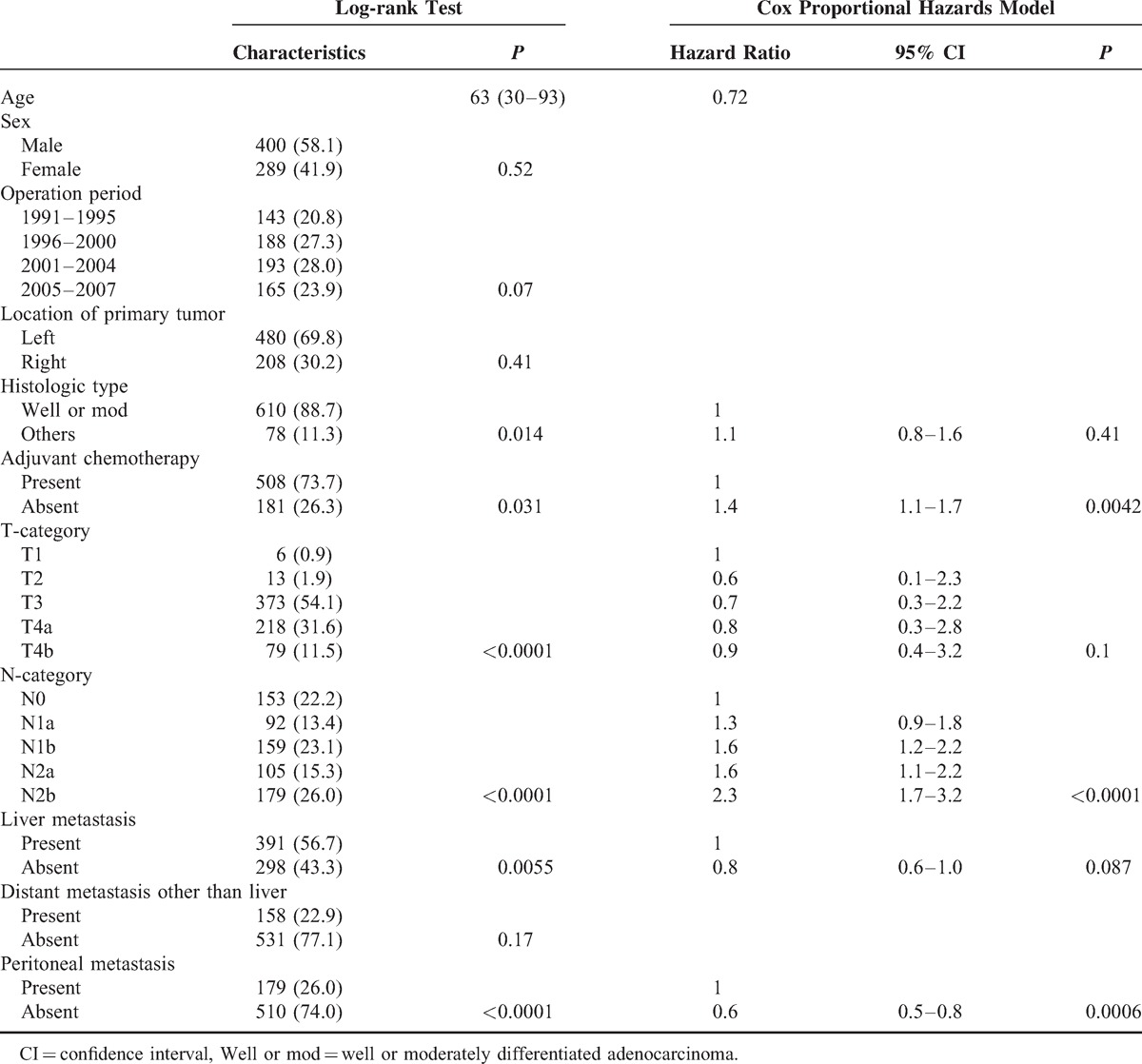
Prognostic Factors of the Entire Cohort (689 Patients)

### Propensity Score Matching Cohort

To calculate the propensity score, a binomial logistic regression model was used. Age (*P* < 0.001), histologic type (*P* = 0.019), and distant metastasis other than liver (*P* = 0.003) were selected. The Hosmer–Lemeshow test showed that this model provided a good fit (*P* = 0.486). The C statistic of this model was 0.63 (95% confidence interval: 0.58–0.67). In this study, 177 patients who received adjuvant chemotherapy were matched with 177 patients who did not receive adjuvant chemotherapy (Table 3). With regard to each predictive parameter, there was no significant difference between patients with and without adjuvant chemotherapy, which showed that these two groups were well matched by propensity score.

**TABLE 3:**
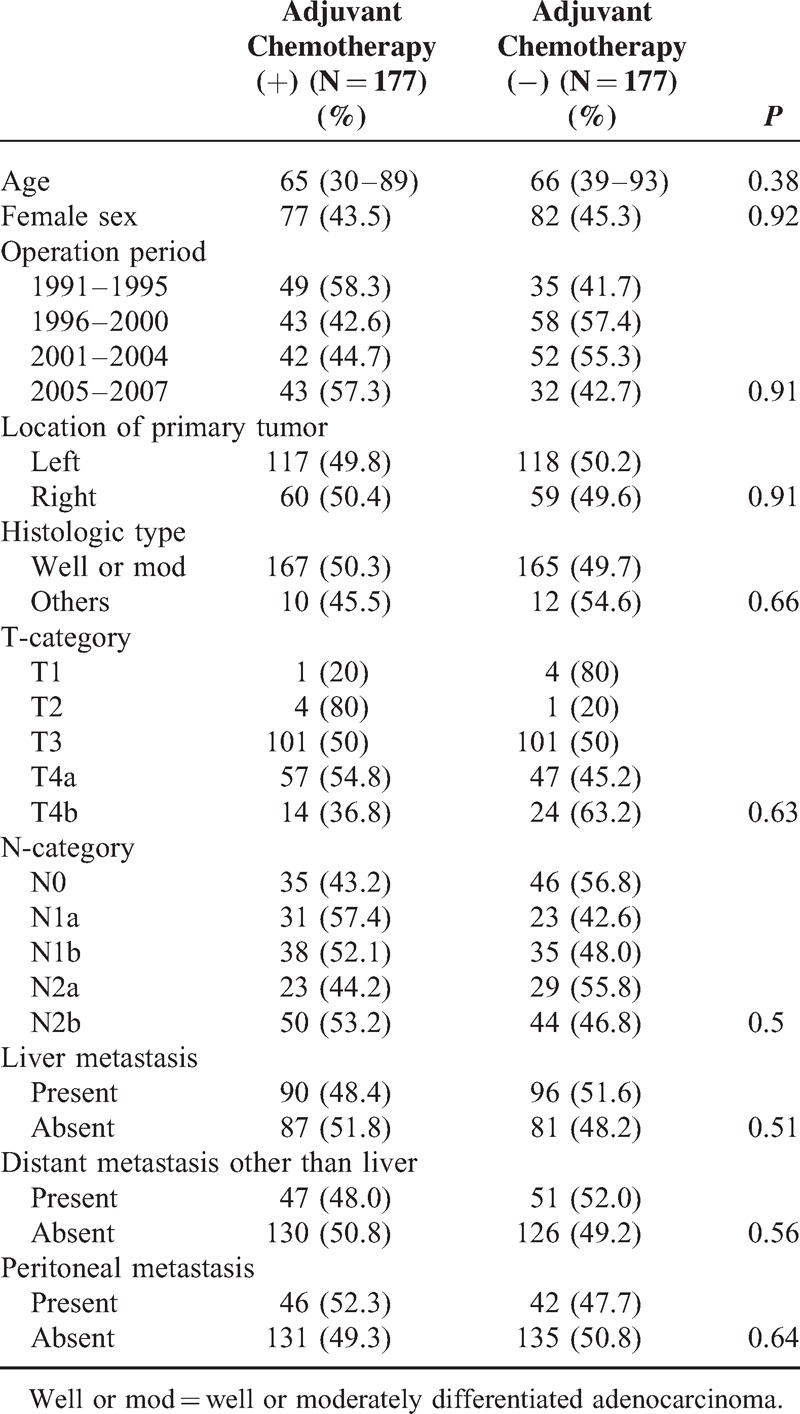
Characteristics of the Propensity Score-matched Cohort (354 Patients)

**FIGURE 2:**
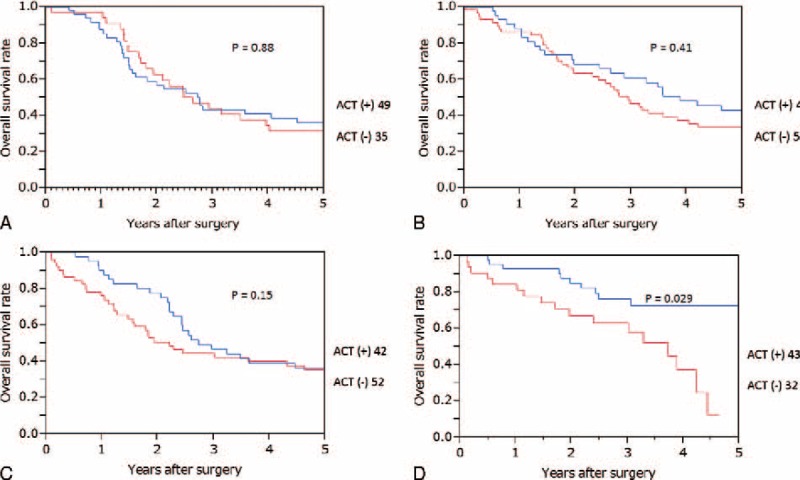
Overall survival curves of patients with and without adjuvant chemotherapy after curative resection for stage IV colorectal cancer by the operation period (A, 1991-1995; B, 1996–2000; C, 2001–2004; and D, 2005–2007). ACT = adjuvant chemotherapy.

In the propensity score-matched cohort, the overall survival of patients with adjuvant chemotherapy was better than that of those without (*P* = 0.026, Figure 1B). When overall survival was compared according to the operation period (1991–1995, Figure 2A; 1996–2000, Figure 2B; 2001–2004, Figure 2C; 2005–2007, Figure 2D), there was a significant difference between patients with and without adjuvant chemotherapy who underwent surgery between 2005 and 2007 (*P* = 0.029, Figure 2D). There were no significant differences in each prognostic parameter between patients with and without adjuvant chemotherapy who underwent surgery between 2005 and 2007 (Table 4).

**TABLE 4:**
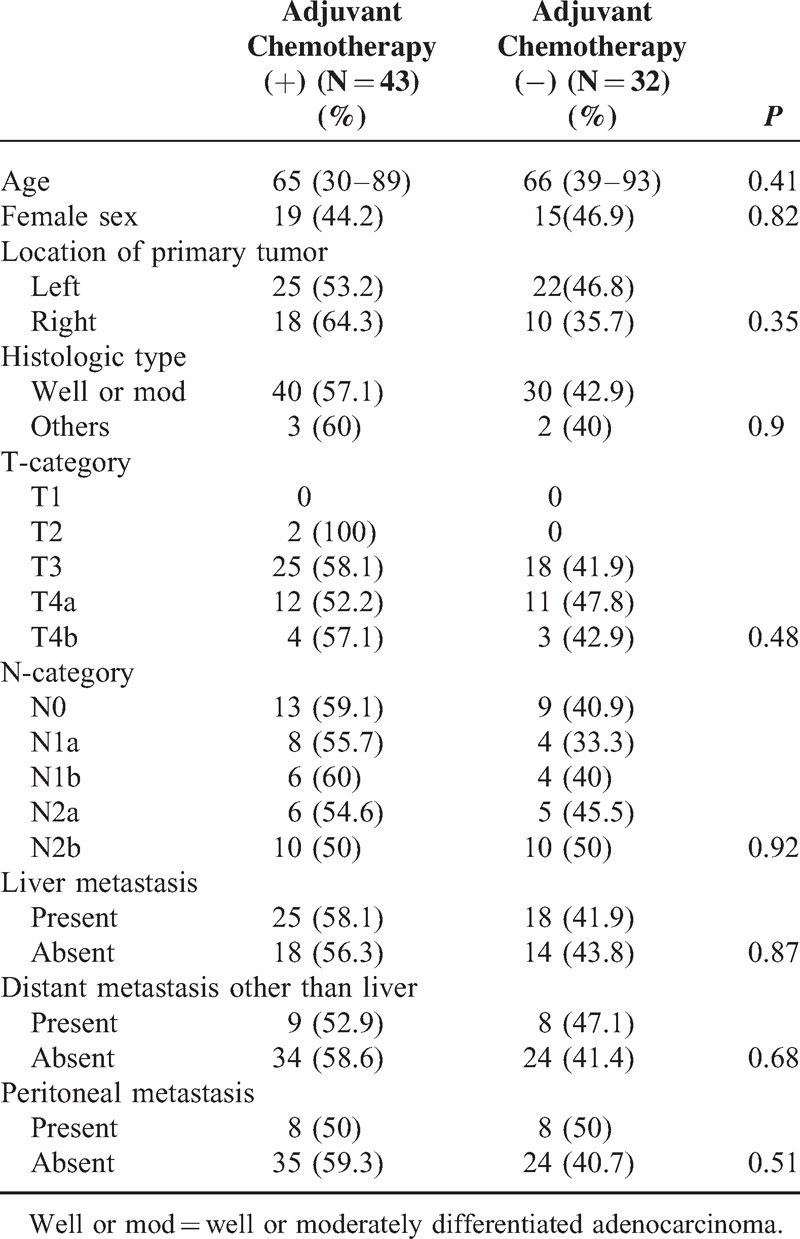
Characteristics of the Patients Treated Between 2005 and 2007 (75 Patients)

## DISCUSSION

The present study demonstrated that adjuvant chemotherapy after curative resection for stage IV colorectal cancer led to better outcomes. The primary endpoint of this study was overall survival because adjuvant chemotherapy after liver metastasis from colorectal cancer led to better disease-free survival, but not to better overall survival in a previous randomized trial and a pooled analysis.^8,9^ In a small-series study, oxaliplatin- or irinotecan-based adjuvant chemotherapy improved both overall and relapse-free survivals in patients after curative resection of synchronous liver metastases from colorectal cancer.^11^ However, another study failed to show the survival benefit of FOLFOX as adjuvant chemotherapy after curative resection for synchronous distant metastases from colorectal cancer.^12^

A number of studies failed to show the survival benefit of adjuvant chemotherapy after curative resection for stage IV colorectal cancer because they failed to reach the target sample size.^8,9,13,14^ The difference in the 5-year survival rate between patients with and without adjuvant chemotherapy after curative resection for liver metastases from colorectal cancer was approximately 10%. In Portier et al's^8^ randomized trial, 5-year overall survival rates of patients with adjuvant chemotherapy and those with surgery alone were 51% and 42%, respectively. In this setting, approximately 460 cases were needed in one-arm with α error of 0.05 and power of 0.8. Even if the primary tumor and distant metastases are resected curatively, such patients have a high risk of recurrence after surgery.^15,16^ Both the National Comprehensive Cancer Network (NCCN) and the European Society for Medical Oncology (ESMO) guidelines recommend adjuvant chemotherapy after curative resection for stage IV colorectal cancer.^17,18^ Under these circumstances, it is difficult to recruit enough patients for a randomized trial to compare the outcomes between patients with adjuvant chemotherapy and those with surgery alone after curative resection for stage IV colorectal cancer.

Although the present study was retrospective, it was possible to collect data from 16 JSCCR member institutions and clarify the clinical benefit of adjuvant chemotherapy after curative resection for both primary tumor and synchronous metastases. As there were originally biases between the adjuvant chemotherapy group and the surgery alone group, a propensity score matching analysis that made the two groups potentially without biases was used. Adjuvant chemotherapy after curative resection for stage IV colorectal improved overall survival using the two matched groups; in particular, the outcomes of patients who underwent surgery in 2005 and later were better than those before 2005. This may be because of the efficacy of oxaliplatin-based adjuvant chemotherapy because oxaliplatin was approved in 2005 in Japan. In fact, only patients who underwent surgery in 2005 or later received FOLFOX as adjuvant chemotherapy. Therefore, a cytotoxic regimen such as FOLFOX might improve overall survival in patients with curative resection for primary colorectal cancer and synchronous distant metastases, although there might be no difference in survival between patients with adjuvant chemotherapy of fluorouracil plus leucovorin and those with surgery alone.

There were some potential limitations in this study. First, as the present study was a retrospective one, there might be biases. We used a propensity score matching analysis to eliminate the biases as much as possible, but the possibility of potential biases remained. For example, data concerning treatments after recurrence were not collected in the present study. Second, the reason for the better outcomes of patients who underwent surgery in 2005 or later might be FOLFOX as chemotherapy after recurrence, as well as FOLFOX as adjuvant chemotherapy. Therefore, a prospective, randomized, controlled trial would be valuable to clarify the definitive usefulness of adjuvant chemotherapy for patients who undergo curative resection for primary colorectal cancer and synchronous distant metastases. In fact, a prospective, randomized, controlled study (JCOG0603) is ongoing to clarify the efficacy of FOLFOX as adjuvant chemotherapy after curative resection for liver metastasis from colorectal cancer.^19^ The results of JCOG0603 are awaited.

## CONCLUSIONS

The present study demonstrated that adjuvant chemotherapy improved overall survival after curative resection for stage IV colorectal cancer. The efficacy of each chemotherapeutic regimen in the adjuvant setting for stage IV colorectal cancer should be clarified in the future.
